# Electron Solvation and the Unique Liquid Structure of a Mixed‐Amine Expanded Metal: The Saturated Li–NH_3_–MeNH_2_ System

**DOI:** 10.1002/anie.201609192

**Published:** 2017-01-10

**Authors:** Andrew G. Seel, Helen Swan, Daniel T. Bowron, Jonathan C. Wasse, Thomas Weller, Peter P. Edwards, Christopher A. Howard, Neal T. Skipper

**Affiliations:** ^1^ Department of Chemistry, Inorganic Chemistry LaboratoryUniversity of OxfordSouth Parks RoadOxfordOX1 3QRUK; ^2^ Department of Physics and AstronomyUniversity College LondonGower StreetLondonWC1E 6BTUK; ^3^ National Nuclear LaboratoryCulham Science CentreAbingdonOX14 3DBUK; ^4^ ISIS Spallation Neutron SourceSTFC Rutherford Appleton Laboratory, ChiltonDidcotOX11 0QXUK

**Keywords:** amines, conducting materials, electron transfer, electronic structure, phase transitions

## Abstract

Metal–amine solutions provide a unique arena in which to study electrons in solution, and to tune the electron density from the extremes of electrolytic through to true metallic behavior. The existence and structure of a new class of concentrated metal‐amine liquid, Li–NH_3_–MeNH_2_, is presented in which the mixed solvent produces a novel type of electron solvation and delocalization that is fundamentally different from either of the constituent systems. NMR, ESR, and neutron diffraction allow the environment of the solvated electron and liquid structure to be precisely interrogated. Unexpectedly it was found that the solution is truly homogeneous and metallic. Equally surprising was the observation of strong longer‐range order in this mixed solvent system. This is despite the heterogeneity of the cation solvation, and it is concluded that the solvated electron itself acts as a structural template. This is a quite remarkable observation, given that the liquid is metallic.

Alkali metals demonstrate an exceptional solubility in NH_3_, yielding intensely colored conducting solutions that have fascinated chemists since the time of Sir Humphry Davy.^1^, ^2^ Varying the concentration of metal in these liquids dramatically alters the electronic, magnetic, and structural properties of the solutions, and enables us to experimentally determine the manner in which liquid systems accommodate excess electron density. At a low concentration of metal/electrons, the solutions are electrolytic, whereby the metal valence electrons have been ionized into solution and exist as solvated electrons propagating between solvent cavities.^3^ Increasing the concentration results in metallization in the liquid phase, which for the Li−NH_3_ system occurs at a mere 4 mol % metal (MPM).^2^ Interestingly at lower temperatures, below *T*_C_=210 K, the point of the Mott‐type metal–insulator transition (MIT) is obscured by a pronounced liquid–liquid phase separation.^2^ This illustrates that the localized and delocalized electron states do not readily co‐exist, which is dramatically manifested by the fact that the more concentrated metallic solution floats above the dilute electrolytic phase for *T*<*T*_c_.^2^, ^4^ Above 8 MPM the solutions do not exhibit phase separation, appearing golden up to the concentration limit of 20 MPM,^5^ the expanded metal Li(NH_3_)_4_.^6^, ^7^ The concentration and temperature dependence of this liquid–liquid phase separation has been mapped through multi‐element NMR spectroscopy.^8^

Along with metal concentration, chemical tunability of the electronic properties of these systems can be achieved by varying the amine. Lithium will also dissolve in MeNH_2_ to yield a system whereby solvated electrons transition to a metallic state.^3^,^9^, ^10^, ^11^, ^12^ The MIT in Li–MeNH_2_ occurs at 15 MPM, a notably higher concentration than in Li–NH_3_. No liquid–liquid phase separation has been detected across the full concentration range of Li–MeNH_2_, and the solution remains a deep blue, albeit with a metallic luster. The conductivity of liquid Li(MeNH_2_)_4_ is 400 Ω^−1^ cm^−1^ (compared to 15 000 Ω^−1^ cm^−1^ in Li(NH_3_)_4_), which is close to Mott's minimum conductivity limit, demonstrating that the Li–MeNH_2_ system lies just on the metallic side of the MIT.

The differing metallic properties of Li–NH_3_ and Li–MeNH_2_ are also reflected in their distinct liquid structures.^13^, ^14^ All metallic metal amines share the trait of being highly structured liquids,^13^, ^14^, ^15^, ^16^ with distinct M_(solv)_–M_(solv)_ correlations. In both Li–NH_3_ and Li–MeNH_2_ solutions, Li is found to be four‐coordinate, which has subsequently been found to be the case in the gas phase,^17^, ^18^, ^19^ and through computational investigation.^1^, ^19^, ^20^, ^21^ The volumetric expansion of these liquid metals across their insulator–metal transition is also accompanied by the appearance of void spaces, which is presumably linked to the conduction electron density. In Li–NH_3_ these voids take the form of channels between Li(NH_3_)_4_ units,^13^ whereas in Li(MeNH_2_)_4_ the voids are spatially isolated.^14^ This is concordant with magnetic measurements that suggest electronic conduction in Li(MeNH_2_)_4_ is via rapid migration of electrons between these polaronic voids, which begin to localize in the solid state.^22^, ^23^

The question then is how a mixture of Li(NH_3_)_4_ and Li(MeNH_2_)_4_ will behave. Surprisingly, perhaps, these mixed amine solutions have only previously been studied by optical absorption towards the limit of infinite dilution.^24^ Herein we report the first studies of the liquid structure of Li‐NH_3_‐MeNH_2_ in the concentrated regime at 20 MPM, an empirical stoichiometry of Li(NH_3_)_2_(MeNH_2_)_2_. We note that after equilibration these solutions appear to the eye as homogenous red/bronze liquids (Figure 1), which upon dilution demonstrate a pronounced liquid–liquid phase separation whereby the lustrous phase floats above a deep‐blue solution. We have observed this phase separation across a very large concentration range of Li, from 2–19 MPM. As confirmation of the homogeneity at 20 MPM, Figure 1 shows that only a single feature is witnessed in the corresponding ^7^Li NMR spectrum. This has proven to be an extremely sensitive indication of homogeneity in previous metal–amine studies.^8^ Interestingly, the ^7^Li peak appears at higher ppm than both Li(NH_3_)_4_ and Li(MeNH_2_)_4_,^8^, ^12^, ^25^ suggesting a higher conduction electron spin‐density at the Li nucleus (or indeed larger molar spin susceptibility) in the mixed amine than in either of the parent amines. The temperature dependence of the ^7^Li Knight shift^8^ and the Dysonian lineshape of the ESR are also indicative of a homogeneous metallic system.^22^, ^26^, ^27^

**Figure 1:**
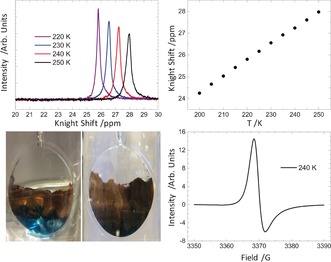
Top left: ^7^Li NMR for 20 MPM Li(NH_3_)_2_(MeNH_2_)_2_ Top right: Temperature dependence of the ^7^Li Knight shift, Bottom left: Homogenization of the 20 MPM system showing initial phase separation (left) and homogenous liquid (right). Bottom right: ESR line shape for 20 MPM Li(NH_3_)_2_(MeNH_2_)_2_.

Neutron diffraction is a uniquely powerful method for determining the liquid structure of metal amines, allowing for a number of isotopically distinct samples to be measured (defined in the Experimental Section below). Figure 2 presents an example fit to an experimental total structure factor, *F*(*Q*), from samples A and H in Table 1. Also shown is the difference spectrum [*F* ${}_{{}^{6}\mathrm{Li}}$ (*Q*)−*F* ${}_{{}^{\mathrm{nat}}\mathrm{Li}}$ (*Q*)] and its Fourier transform, the Li‐centered total pair correlation function, Δ*G*_Li_(*r*). The single principal peak at 1.75 Å^−1^, and importantly the existence of a sharp pre‐peak at 0.97 Å^−1^, attest to the homogeneity of the 20 MPM solution. The pre‐peak is a structural feature witnessed in solvated‐electron systems as they transition to the metallic state, and arises owing to strong intermediate range ordering in the liquid.^13^, ^14^, ^15^ Integration of the principal peak in the Δ*G*_Li_(*r*) distribution corresponds to the coordination number of Li, and is consistent with approximately 4 solvent molecules, as reported previously for other Li–RNH_2_ systems.^13^, ^14^

**Figure 2:**
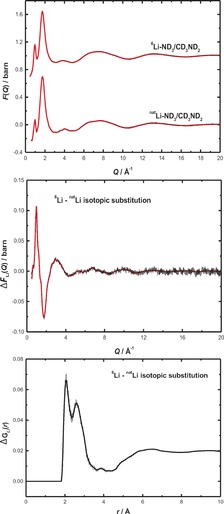
Top: Experimental total structure factors for 20 MPM ^6^Li–ND_3_–CD_3_ND_2_ and ^nat^Li–ND_3_–CD_3_ND_2_ solutions. Middle: First‐order Li difference structure factor. Bottom: Corresponding Li partial pair correlation.

**Table 1:** Isotopic substitutions in samples of 20 MPM Li–NH_3_–MeNH_2_.

Sample	Li	NH_3_	MeNH_2_
A	^nat^Li	ND_3_	CD_3_ND_2_
B	^nat^Li	NH_3_	CD_3_NH_2_
C	^nat^Li	[NH_3_:ND_3_]	[CD_3_NH_2_:CD_3_ND_2_]
D	^nat^Li	ND_3_	CH_3_ND_2_
E	^nat^Li	ND_3_	[CD_3_ND_2_:CH_3_ND_2_]
F	^nat^Li	NH_3_	CH_3_NH_2_
G	^nat^Li	[NH_3_:ND_3_]	[CD_3_ND_2_:CH_3_NH_2_:CD_3_NH_2_:CH_3_ND_2_]
H	^7^Li	ND_3_	CD_3_ND_2_

To determine the spatial arrangement of lithium and amine species in the system, the empirical potential structure refinement (EPSR) technique was invoked to produce a structural model that was refined simultaneously to all the isotopically unique experimental *F*(*Q*) functions (Table 1).^28^

Figure 3 shows the EPSR fits to the experimental data, with the EPSR model fitting well the experimentally obtained partial structure factors. The corresponding real‐space distribution of solvent molecules around the lithium ions is given in Figure 4. The Li−N distance for both NH_3_ and MeNH_2_ is about 2.0 Å, in close agreement with that observed for both Li(NH_3_)_4_ and Li(MeNH_2_)_4_. Integration of this first solvation shell gives an average co‐ordination number of 2.01 and 1.78 for Li–MeNH_2_ and Li–NH_3_, respectively. The orientational distribution of the coordinated solvent about Li can be extracted from the EPSR model (Figure 4 a–d). Only a small distortion from tetrahedral geometry for a given Li_(solv)_ unit is found, with the MeNH_2_ ligands acting to compress the tetrahedra slightly along the tetrahedral *C*_2_ axis. The dipole moment of NH_3_ is orientated directly towards the cation, whereas the Li–MeNH_2_ angle is about 109°. The relative orientation of neighboring Li_(solv)_ units is not isotropic, with the vertex (that is, the NH_3_/MeNH_2_ molecules) of one tetrahedral unit approaching the face and edges of the adjacent tetrahedral unit.

**Figure 3:**
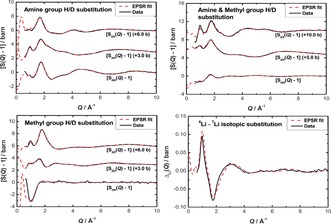
Experimental (black) and modeled (red) partial structure factors for 20 MPM Li–NH_3_–MeNH_2_.

**Figure 4:**
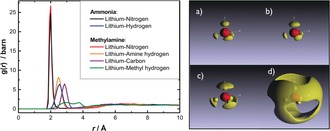
Left: Li‐centered partial distribution functions for 20 MPM Li–NH_3_–MeNH_2_. Right: Li‐centered three‐dimensional configurations for radial range *r*=1.5–2.5 Å, 80 % probabilities: a) Li−N (NH_3_), b) Li−N (MeNH_2_), c) Li‐N‐C (MeNH_2_), d) Li−N *r*=2.5–6.0 Å.

Importantly, the EPSR model also reveals the statistical distribution of ammonia and methylamine molecules in the [Li(NH_3_)_*n*_(MeNH_2_)_*m*_]^+^ tetrahedral complexes (Supporting Information, Figure S2). This distribution shows that for around 40 % of cations (*n*,*m*) is (2,2), for around 50 % it is (1,3) or (3,1), and for the remaining 10 % it is (0,4) or (4,0). The cation solvation is therefore significantly heterogeneous, and it is all the more surprising that we observe a sharp diffraction pre‐peak in our data, indicative of longer‐range order in the liquid. In the absence of a single cationic motif, we must conclude that the solvated electron itself acts as a structural template that drives homogenization. This hypothesis is consistent our observation of a single ^7^Li Knight shift (Figure 1), and is truly remarkable given that the system is metallic.

The manner in which excess electrons are accommodated can be determined by examining the liquid structures for void regions: spherical volumes with circa 2.5 Å radii containing no atoms. Previous studies have demonstrated the existence of these regions in each of the metallic metal amines, with stark differences in their size and orientation, which correlate with their differing metallic properties. In the highly conducting Li–NH_3_ liquid these voids are centered above the protons of the NH_3_, forming channels between solvated Li(NH_3_)_4_ units (hence the moniker expanded metal). In the poor metal, Li–MeNH_2_, the cavities are spatially isolated from one another, removed from the primary solvation environment of Li and bounded by the methyl groups of coordinated MeNH_2_. The void–void distribution function in our mixed amine solution is presented in the Supporting Information, Figure S3, and shows weak inter‐void correlations beyond around 4 Å.

Figure 5 shows the orientation of void regions relative to an NH_3_ and MeNH_2_ molecule in the 20 MPM Li–NH_2_–MeNH_2_ system. It is seen that the voids (and conduction electron density) lie around the circumference of NH_3_ and above the amine group of MeNH_2_. This structure is distinctly different to either the Li–NH_3_ or Li–MeNH_2_ system, and indicates that the electron density is drawn closer to Li, around the circumference of the solvating NH_3_/MeNH_2_ molecules.

**Figure 5:**
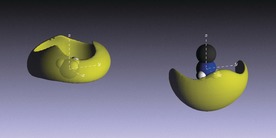
Void regions relative to the NH_3_ (left) and MeNH_2_ groups (right) in 20 MPM Li–NH_3_–MeNH_2_. *r*=2.4–5.4 Å.

In conclusion, we have discovered that the mixed amine system Li(NH_3_)_2_(MeNH_2_)_2_ forms a highly structured homogenous liquid metal. Each cation is tetrahedrally coordinated by approximately four solvent molecules, but there is significant heterogeneity in the NH_3_:MeNH_2_ ratio around individual Li^+^ ions. This lack of a single cationic motif makes our observation of strong intermediate‐range order in the solutions particularly surprising. Even though the system is metallic, we therefore turn to the solvated electron as a potential structural template. Indeed, we find that the system appears unique amongst current expanded metal–amine solutions for the manner in which the excess electron density is accommodated. This is drawn closer to the lithium cations than in either the constituent lithium–ammonia or lithium–methylamine solutions, and we observe only a single Knight shift on the lithium nuclei. Mixed‐solvent metal–amine solutions therefore present a new set of challenges for our understanding of electron solvation, and the nature of the concomitant phase separation of the localized and delocalized electronic states.

## Experimental Section

H/D and ^7^Li/^nat^Li isotopic substitution experiments (where nat denotes natural abundance) were performed using the SANDALS diffractometer at the ISIS Spallation Neutron and Muon Source, UK.^29^ A clean sample of Li metal was loaded under argon into a sealed flat‐plate null‐coherent scattering Ti/Zr container, with sample and wall thicknesses of 1 mm. The container and sample were attached to a stainless steel gas‐rig and evacuated to 10^−5^ mbar. To prepare a sample of 20 MPM Li–NH_3_–MeNH_2_, an equimolar ratio of NH_3_:MeNH_2_ were premixed to the exact volume required, and cryo‐pumped onto the Li sample at 60 K. This avoided any problem in the different boiling temperatures of the amines. The sample was then isolated and warmed to 240 K. The samples were monitored for Bragg reflections in the warming process to ensure that the solutions were fully homogenized. Data were collected for a period of 8 h, and spectra were corrected for background, multiple scattering and absorption following standard protocols for SANDALS data.

The measured total structure factor for a given system comprising *n* chemical species can be expressed as in Eq. (1):

(1) F(Q)=∑nα=1∑nβ=1cαcβbαbβ[Sαβ(Q)-1]

where *c_i_* denotes the atomic fraction of species *i* and *b_i_* is the bound coherent neutron scattering length for that species. *S*_*αβ*_(*Q*) is the Faber–Ziman partial structure factor, and *Q* the scattering vector. The detailed method for extraction the partial structure factor from isotopically varying *F*(*Q*) values is detailed elsewhere. *F*(*Q*) is the Fourier transform of the total pair correlation function *G*(*r*). The specific isotopic substitutions for prepared samples are given in Table 1.

The empirical potential structure refinement (EPSR) method was used to model the measured neutron total scattering data.^28^ A structural model comprising 2000 species (Li and molecular NH_3_, MeNH_2_) is simultaneously refined to each of the isotopically unique experimental *F*(*Q*) spectra (samples A–H in Table 1), enabling a rigorous constraint to the resultant structural model, and extraction of atom specific pair‐correlation functions, *G*_*αβ*_(*r*). The inter‐atom potentials in EPSR are comprised of a Lennard‐Jones potential and Coulombic term, and is iterated throughout the simulation until a suitable convergence to experimental *F*(*Q*) spectra is reached. The seed potentials used in this work were the same as used previously for metal–amine liquid structure studies.^12^, ^13^, ^14^, ^15^

NMR spectra were recorded on a Bruker AVIIIHD 400 MHz spectrometer, referenced to LiCl in D_2_O. ESR was performed at X‐band frequencies on a Bruker EMXmicro spectrometer. Samples for each were prepared in the manner outlined above, within spectrosil quartz tubes that were subsequently sealed. All handling and transfer of samples was conducted under cryogenic conditions.

## Conflict of interest

The authors declare no conflict of interest.

## Supporting information

As a service to our authors and readers, this journal provides supporting information supplied by the authors. Such materials are peer reviewed and may be re‐organized for online delivery, but are not copy‐edited or typeset. Technical support issues arising from supporting information (other than missing files) should be addressed to the authors.

Supplementary
