# Supplementary material for: Electron Solvation and the Unique Liquid Structure of a Mixed‐Amine Expanded Metal: The Saturated Li–NH3–MeNH2 System
Source: Angew Chem Int Ed Engl. 2017 Jan 10;56(6):1561–5. doi: 10.1002/anie.201609192 (PMC5396365; doi:10.1002/anie.201609192)
Supplement: Supplementary file 1 — Supplementary [file ANIE-56-1561-s001.pdf]

Angewandte Chemie
Eine Zeitschrift der Gesellschaft Deutscher Chemiker

Supporting Information

Electron Solvation and the Unique Liquid Structure of a Mixed-Amine Expanded Metal: The Saturated Li–NH_3–$MeNH_2$ System

*Andrew G. Seel, Helen Swan, Daniel T. Bowron, Jonathan C. Wasse, Thomas Weller, Peter P. Edwards, Christopher A. Howard, and Neal T. Skipper**

anie_201609192_sm_miscellaneous_information.pdf

WILEY-VCH

Supporting Information

Table of Contents

Figure S1: Expanded view of Figure 1c, showing equilibration and homogenisation of the 20 MPM $Li(NH_3)_2(MeNH_2)_2$ system on going from left to right (Page S2).

Figure S2: Void-void radial distribution function in 20 MPM $Li(NH_3)_2(MeNH_2)_2$ obtained from neutron diffraction in conjunction with Empirical Potential Structure Refinement (EPSR). Main: The radial distributions of void space surrounding a given point at the centre of a sphere of void volume of 2.5 Å radius. The large intensity below approximately 4 Å arises from intra-void correlations caused by the non-spherical nature of the void regions. Insert: The radial distribution function with contributions from the intra-void correlations removed, demonstrating correlated inter-void distances beyond 4 Å (Page S3).

Figure S3: Coordination about Li in 20 MPM $Li(NH_3)_2(MeNH_2)_2$ solution as obtained from neutron diffraction in conjunction with Empirical Potential Structure Refinement (EPSR), demonstrating the statistical distribution of NH_3 and $MeNH_2$ in the Li(NH3)n(MeNH2)m+ tetrahedral complexes (Page S4).

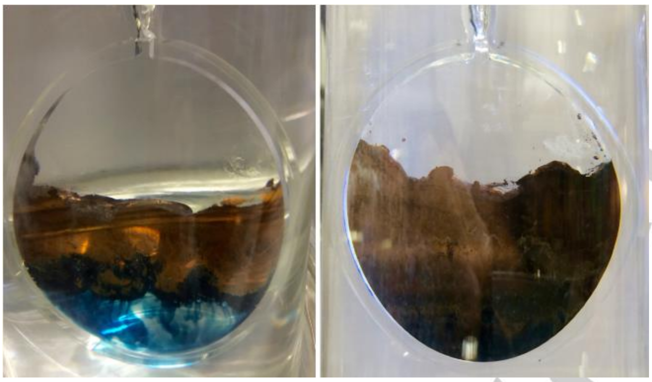

Figure S1. Expanded view of Figure 1c, showing equilibration and homogenisation of the 20 MPM $Li(NH_3)_2(MeNH_2)_2$ system on going from left to right.

WILEY-VCH

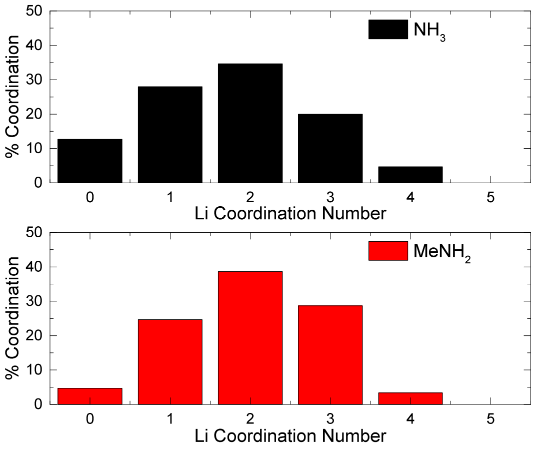

Figure S2. Coordination about Li in 20 MPM $Li(NH_3)_2(MeNH_2)_2$ solution as obtained from neutron diffraction in conjunction with Empirical Potential Structure Refinement (EPSR), demonstrating the statistical distribution of NH_3 and $MeNH_2$ in the $[Li(NH_3)_n(MeNH_2)_m]^+$ tetrahedral complexes.

WILEY-VCH

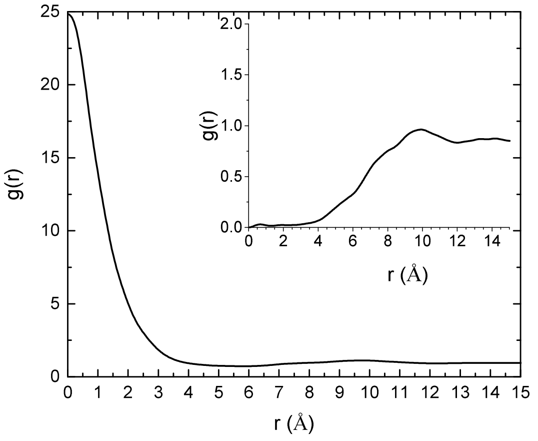

Figure S3. Void-void radial distribution function in 20 MPM $Li(NH_3)_2(MeNH_2)_2$ obtained from neutron diffraction in conjunction with Empirical Potential Structure Refinement (EPSR). *Main*: The radial distributions of void space surrounding a given point at the centre of a sphere of void volume of 2.5 Å radius. The large intensity below approximately 4 Å arises from intra-void correlations caused by the non-spherical nature of the void regions. *Insert*: The radial distribution function with contributions from the intra-void correlations removed, demonstrating correlated inter-void distances beyond 4 Å.

WILEY-VCH
